# Poplar Bud (*Populus*) Extraction and Chinese Propolis Counteract Oxidative Stress in *Caenorhabditis elegans* via Insulin/IGF-1 Signaling Pathway

**DOI:** 10.3390/antiox13070860

**Published:** 2024-07-18

**Authors:** Shuo Wang, Chengchao Yang, Yaling Luo, Qingyi Chen, Mengyang Xu, Yuntao Ji, Xiasen Jiang, Changqing Qu

**Affiliations:** 1 Engineering Technology Research Center of Anti-Aging Chinese Herbal Medicine of Anhui Province, Biology and Food Engineering School, Fuyang Normal University, Fuyang 236000, China; 2 Liaoning Provincial Institute of Poplar, Gaizhou 115200, China

**Keywords:** poplar bud extracts, Chinese propolis, *Caenorhabditis elegans*, oxidative stress, insulin/IGF-1 signaling pathway

## Abstract

Poplar buds are characterized by a high content of phenolic compounds, which exhibit a broad spectrum of biological activities. However, the relationship between Chinese propolis and poplar buds based on their antioxidant capacities and underlying mechanisms remains unclear. This study aimed to investigate the antioxidant properties of poplar bud (*Populus*) extract (PBE) and Chinese propolis (CP) and to elucidate the mechanisms behind their activity. High-performance liquid chromatography (HPLC) analysis revealed that both PBE and CP contain a significant amount of phenolic acids and flavonoids. 2,2-diphenyl-1-picrylhydrazyl (DPPH), 2,2′-azino-bis (3-ethylbenzothiazoline-6-sulfonic acid) (ABTS), and ferric-reducing antioxidant power (FRAP) assays demonstrated that PBE and CP possess excellent antioxidant activity. Furthermore, administration of PBE and CP improved the survival rate of *C. elegans* under oxidative stress. They also decreased the levels of reactive oxygen species (ROS) and malondialdehyde (MDA), while enhancing the activity of antioxidant enzymes (SOD, CAT). PBE and CP intervention upregulated the expression of key genes *daf-16*, *sod-3*, *hsp-16.2*, and *skn-1* in nematodes. This suggests that the antioxidant activity of PBE and CP is dependent on *daf-16* and *skn-1* signaling pathways. In conclusion, poplar bud extracts ha have the potential to become a substitute for propolis and a potential therapeutic agent for treating diseases associated with oxidative damage.

## 1. Introduction

Propolis, a resinous waxy material, is collected by honeybees which combine secretions from plant buds, leaves, or sap flows with their saliva and beeswax [1]. This complex substance contains phenolic acids, flavonoids, and alkenes [2,3]. Prized for its high value, propolis exhibits a range of pharmacological activities, including antioxidant, anti-inflammatory, hypoglycemic, and anticancer properties, as demonstrated by modern research [4,5]. However, limitations in propolis production coupled with high demand necessitate the exploration of alternative sources. Poplar trees, particularly the resin from *Populus* species, are a primary source of propolis and contribute significantly to its bioactive compounds [6]. Numerous studies have shown that the active components of propolis are largely found in poplar buds. Additionally, research suggests that the biological activities observed in propolis are primarily attributed to the plant-based ingredients collected by bees [7,8]. This finding further strengthens the hypothesis that poplar buds possess pharmacological effects similar to those of propolis.

Poplar species undoubtedly constitute the primary source of resin utilized by honeybees. However, due to their wide geographic range and diverse botanical preferences, bees collect resin from a variety of plant sources [9]. Consequently, propolis exhibits significant variations in both chemical composition and biological activity [10]. In contrast, poplar buds are typically harvested from a specific tree species within a defined geographic area. This difference in collection methods raises questions regarding the precise overlap in compounds between poplar buds and propolis [11,12]. Gas chromatography–mass spectrometry (GC–MS) analysis of *Populus alba*, *Populus nigra*, and *Populus tremuloides* bud extracts alongside propolis samples from the same region revealed the presence of aromatic acids, flavones, chalcones, terpenes, and fatty acids in both materials. However, the specific content of these components differed slightly [11]. Some studies have reported the presence of salicin and catechol in poplar buds, while these compounds are absent in propolis [12,13]. Studies employing 2,2-diphenyl-1-picrylhydrazyl (DPPH), 2,2′-azino-bis (3-ethylbenzothiazoline-6-sulfonic acid) (ABTS), and ferric-reducing antioxidant power (FRAP) assays have shown that *Populus balsamifera* bud extracts possess superior antioxidant activity compared to propolis extracts obtained from the same region [11]. However, contrasting results have emerged from research conducted in China. It has been reported that propolis exhibits a higher phenolic content and stronger antioxidant activity compared to poplar bud extracts [4,14]. Historically, research on poplar buds primarily focused on differentiating them from propolis, with less emphasis placed on their potential pharmacological activities [15]. Conversely, numerous studies have investigated the antioxidant properties of propolis utilizing cell and animal models. Despite these advancements, the precise relationships between the antioxidant capacities of propolis and poplar buds and the underlying molecular mechanisms remain to be elucidated.

To determine the content of flavonoids and phenolic acids, we employed high-performance liquid chromatography (HPLC) to analyze the phytochemical composition of Chinese propolis and poplar bud extract. We then evaluated the in vitro antioxidant activity of poplar bud (*Populus*) extract (PBE) and Chinese propolis (CP) using DPPH, ABTS, and FRAP methods. Additionally, an in vivo nematode model of oxidative stress was established to assess the antioxidant activity of PBE and CP. To elucidate the underlying mechanisms of PBE and CP’s antioxidant activity, screening was performed using transgenic nematodes. Furthermore, RT-PCR was employed to detect the expression of antioxidant genes. These investigations provide a foundation for the development and utilization of poplar bud resources.

## 2. Materials and Methods

### 2.1. Materials and Chemicals

All materials were stored in the laboratory, including Chinese propolis, poplar buds, *Escherichia coli* OP50, and various *C. elegans* strains. The Chinese propolis used in this study was harvested in March 2023 from Dezhou, Shandong Province, China, by local beekeepers. Poplar buds were collected from *Populus* trees near the bee farm. Commercial assay kits for measuring catalase (CAT), malondialdehyde (MDA), superoxide dismutase (SOD), and total protein content were purchased from Solarbio (Beijing, China). Caffeic acid phenethyl ester, ferulic acid, isoferulic acid, apigenin, naringenin, caffeic acid, *p*-coumaric acid, 3,4-dimethoxycinnamic acid, galangin, quercetin, chrysin, pinocembrin, kaempferol, and DPPH were obtained from Sigma-Aldrich (St. Louis, MO, USA). Pinobanksin and benzyl caffeate were purchased from Ningbo Haishu Apexocean Biochemicals Co., Ltd. (Ningbo, Zhejiang, China). Analytical grade methanol, 2′,7′-dichlorofiuorescin diacetate (H_2_DCFDA), and glacial acetic acid were obtained from MREDA. Finally, 5-fluoro-2′-deoxyuridine (FUDR), dimethyl sulfoxide, and juglone were all of analytical grade.

### 2.2. Sample Preparation and Phytochemical Profile Analysis by HPLC

Sample preparation: Poplar buds were prepared for analysis by soaking them in 75% ethanol overnight at a 1:10 material-to-liquid ratio. As described previously [4], the extract was evaporated at 55 °C in a rotary evaporator and concentrated under reduced pressure. Subsequently, the samples were dried in a 55 °C oven until a constant weight was achieved. The resulting PBE and CP were stored at −20 °C.

An analysis of phytochemicals in PBE and CP was performed using HPLC. Prior to injection, both PBE and CP were redissolved in anhydrous ethanol at a concentration of 5 mg/mL. The HPLC system utilized a Sepax C18 column (4.6 × 250 mm, 5 μm) maintained at 33 °C. Samples (5 μL) were first passed through 0.45 μm filters for purification before injection through an automatic sampler system. Detection was performed at 280 nm. A gradient mobile phase consisting of solvent A (0.1% glacial acetic acid in ultrapure water) and solvent B (methyl alcohol) was employed. The gradient profile was as follows: 0–30 min (15–35% B), 30–46 min (35–44% B), 46–70 min (44–50% B), 70–77 min (50–52% B), 77–92 min (52–60% B), 92–115 min (60–75% B), 115–125 min (75–95% B), and 125–135 min (95–15% B) at a flow rate of 1 mL/min.

### 2.3. Determination of Total Phenolic Acid and Flavonoid Contents

The phenolic acid content in the samples was determined using the Folin–Ciocalteu assay [16]. Briefly, 1 mL of Folin–Ciocalteu reagent was mixed with 0–0.8 mL of 0.1 mg/mL gallic acid solution. Subsequently, 5 mL of 1 M Na_2_CO_3_ solution was added, and the final volume was adjusted to 10 mL with distilled water. The reaction mixture was incubated in the dark at room temperature for 1 h. The absorbance of the solution was then measured at 760 nm. A gallic acid standard curve was used to express the phenolic content as gallic acid equivalents.

The total flavonoid content was determined according to the method described by Jiang et al. [16]. Briefly, the samples were mixed with solutions of 1 M AlCl_3_ and C_2_H_3_KO_2_. Following incubation in the dark at room temperature for 1 h, the absorbance of the mixture was measured at 415 nm. Rutin was used as a standard, with the flavonoid content expressed as rutin equivalents (mg/g).

### 2.4. In Vitro Antioxidant Test

(1) DPPH scavenging activity

DPPH-radical-scavenging activity was determined following the methods of Zhou et al. [17] with minor modifications. The sample concentrations used were 20–180 μg/mL, while vitamin C (VC) (used as the standard solution) was employed at concentrations of 2–36 μg/mL. The reaction mixture consisted of DPPH solution (1 mg/mL) mixed with the sample (A1). A control reaction (A2) was prepared by reacting DPPH solution with anhydrous ethanol. Following a 30 min incubation period in darkness at room temperature, the absorbance values were measured at 517 nm. The DPPH-scavenging capacity was then calculated using the following formula:

Scavenging activity (%) = [(A2 − A1)/A2] × 100%(1)

(2) ABTS scavenging activity

The ABTS-radical-scavenging activity of PBE and CP was determined to evaluate their in vitro antioxidant activity [18]. Briefly, equal volumes of ABTS solution (7 mmol/L) and potassium persulfate solution (140 mmol/L) were mixed and allowed to react in the dark for 16 h at room temperature. The resulting solution was then diluted with ethanol to achieve an absorbance of approximately 0.7 at 732 nm. PBE and CP were prepared in ethanol at varying concentrations ranging from 0 to 140 μg/mL. Three reaction mixtures were prepared for each sample concentration: (A1) containing 0.2 mL of sample and 0.4 mL of ABTS solution, (A2) containing 0.2 mL of sample and 0.4 mL of anhydrous ethanol, and (A3) containing 0.2 mL of anhydrous ethanol and 0.4 mL of ABTS solution. All the mixtures were mixed thoroughly and incubated in the dark for 10 min at room temperature. Finally, the absorbance of each mixture was measured at 732 nm. VC at different concentrations served as a positive control. The ABTS-scavenging capacity (%) was calculated using the following formula:

Scavenging activity (%) = [1 − (A1 − A2)/A3] × 100%(2)

(3) FRAP assay

The FRAP assay was also employed to assess the antioxidant activities of PBE and CP [19]. An amount of 1 mL of each sample at various concentrations was mixed with 2.5 mL of phosphate buffer (0.2 mol/L, pH 6.6) and 2.5 mL of a 10 mg/mL K_3_[Fe (CN)_6_] solution. This mixture was then incubated at 50 °C for 20 zmin. Following incubation, 2.5 mL of 10% trichloroacetic acid was added, and the mixture was allowed to react in the dark for 10 min at room temperature. Subsequently, 2.5 mL of the supernatant was collected and mixed with 2.5 mL each of 1 mg/mL FeCl_3_ solution and distilled water. After a 10 min incubation, the absorbance of the final mixture was measured at 700 nm. Higher absorbance values correspond to greater total reducing power.

### 2.5. C. elegans Strains and Culture Conditions

*C. elegans* strains used in this experiment were as follows: N2 (wild-type), TJ356:zIs356 [*daf-16*::GFP], CF1553:muIs84 [(pAD76)*sod-3p*::GFP+*rol6* (su1006)], TJ375:gpIs1 [*hsp-16.2*::GFP], LD1: 1dIs7[*skn-1b/c*::GFP+*rol-6* (su1006)], and CL2166:dvIs19 [(pAF15) *gst-4p*::GFP::NLS] III. Nematodes and *E. coli* OP50 were purchased from the *Caenorhabditis* Genetic Center (CGC) (Minneapolis, MI, USA). All *C. elegans* strains were cultured in nematode growth media (NGM) supplemented with *E. coli* OP50 at 20 °C. Synchronized nematodes were obtained according to the method described by Li et al. [20].

### 2.6. Toxicity Test

Mild stress induces adaptive cellular responses that prepare for subsequent severe stress, a phenomenon known as hormonal effects [21]. Hormesis describes the situation where an active ingredient produces positive effects at low doses and harmful effects at high doses. Therefore, the choice of dosage is crucial [22]. PBE or CP was dissolved in DMSO and then mixed with NGM media cooled to 55 °C to give concentrations of 20, 40, 80, 160, and 320 μg/mL. DMSO concentration in NGM was 0.2%. For comparison, a negative control containing 0.2% DMSO and a positive control containing 36 μg/mL rutin (dissolved in DMSO) were also included in this study. Synchronized N2 nematodes (L4 stage) were washed three times with M9 solution and then collected. The nematodes were subsequently transferred to NGM plates. After 3 days of incubation, the survival rate of the nematodes on each plate was determined using a stereomicroscope.

### 2.7. Body Length and Body Width Assay

Synchronized L4 stage N2 nematodes were transferred to NGM media supplemented with or without PBE and CP for 3 days. To avoid the generation of progeny, experiments were performed using NGM plates containing 50 μmol/L FUDR. Following a wash with M9 buffer, the nematodes were mounted on agarose pads, anesthetized with levamisole hydrochloride, and observed under a microscope. Worms were photographed using a microscope (Olympus, Japan), and their body length and width were analyzed using ImageJ 8.0 software. Each experiment was replicated at least three times with over 20 nematodes per replicate. According to standard methods, the body length of a nematode is defined as the measurement from head to tail, and body width is the widest part of the worm’s body [23].

### 2.8. Progeny Assay

Synchronized L4 stage N2 nematodes were transferred to NGM media containing different concentrations of PBE or CP and incubated at 20 °C for 24 h. During the breeding season, the worms were transferred to new NGM plates daily to differentiate between parents and offspring. A microscope was used to observe and record the number of progenies each day until the nematodes stopped laying eggs: 1 nematode per plate, 10 nematodes in a group.

### 2.9. Oxidative Stress Assay

The oxidative stress resistance assay was performed based on the method described by Tao et al. [24] with some modifications. Synchronized L4 stage N2 nematodes were cultured on NGM plates containing PBE or CP for 3 days at 20 °C. Following this incubation, thirty nematodes were randomly selected and transferred to NGM plates containing 200 μmol/L juglone. The survival rate of the nematodes was then monitored after 8 h. This experiment was repeated three times.

### 2.10. Fluorescence Imaging

The nematode strains TJ356, TJ375, CL2166, LD1, and CF1553 were used to investigate the expression of DAF-16, HSP-16.2, GST-4, SKN-1, and SOD-3, respectively. Fluorescence microscopy (Olympus, Japan) was employed to observe the worms, with at least 30 individuals examined per group. For all strains, *C. elegans* images were taken using a 10× objective. CF1553, LD1, and CL2166 were measured for fluorescence intensity throughout the whole worm. ImageJ software was then utilized to quantify the fluorescence intensity. We quantitatively analyzed the expression of HSP-16.2::GFP in the pharyngeal bulb of TJ375 *C. elegans*. TJ356 *C. elegans* were classified into three categories based on the dominant localization of the DAF-16::GFP fusion protein: “nuclear”, “cytosolic”, and “intermediate”. Worms with more than 20 nuclear fluorescence dots were categorized as nuclear, while those without nuclear fluorescence dots were categorized as cytosolic. Worms in the mid-state were counted as intermediate [25].

### 2.11. ROS Accumulation Assay

Nematode ROS levels were measured as previously described with minor modifications [24]. Briefly, N2 *C. elegans* (L4 stage) were treated with either PBE or CP at 20 °C for 3 days. Following treatment, the nematodes were washed three times with M9 buffer and then incubated with 50 mmol/L H_2_DCFDA at 37 °C for 1.5 h. Finally, the nematodes were immobilized on 2% agarose pads and photographed under a fluorescence microscope. Fluorescence images were collected with constant exposure time in the DAPI channel. ImageJ software was used to quantify the relative fluorescence levels of ROS in each nematode. This software was used to measure fluorescence intensity and remove background signal. Use the polygon tool to draw the overall outline of the object and use integrated density to estimate relative fluorescence intensity.

### 2.12. Determination of SOD, CAT, and MDA

L4 stage N2 *C. elegans* (approximately 500–1000 per group) were treated with PBE or CP for 3 days and collected using M9 buffer. After the nematodes were ultrasonically broken on ice, the protein concentration was determined with a BCA kit. Therefore, the CAT activity, SOD activity, and MDA content were detected with the corresponding assay kits (Solarbio, Beijing, China) following the manufacturer’s instructions.

### 2.13. Quantitative RT-PCR

Total RNA was extracted from L4 stage N2 nematodes (more than 500) per group using a total RNA extraction reagent and was reverse-transcribed to cDNA using a HiScript II 1st Strand cDNA Synthesis Kit (+gDNA wiper) (Vazyme, Nanjing, China). Gene expression was detected using the AceO Universal SYBR gPCR Master Mix and a CFX Connect Real-Time System. The primer sequences are shown in Table 1. Actin-1 (*act-1*) was used as an internal reference, and the data were analyzed by the 2^−ΔΔCt^ method.

### 2.14. Statistical Analysis

Statistical analysis of the data was performed using SPSS 26.0 software. Data visualization was conducted with GraphPad Prism 9.0 software. A one-way ANOVA and unpaired Student’s *t*-tests were employed to assess statistical significance. Results are expressed as mean ± SD. NS denotes no significant difference compared to the control group. Differences compared to the control group were considered significant at *p* < 0.05 (*), *p* < 0.01 (**), *p* < 0.001 (***), and *p* < 0.0001 (****).

## 3. Results

### 3.1. Detection of Phytochemicals in PBE and CP

The contents of phenolic acids and flavonoids in PBE and CP were analyzed, and the results are presented in Table 2. PBE exhibited a phenolic acid content of 234.18 ± 0.95 mg gallic acid eq/g, while CP contained a significantly higher amount (265.77 ± 2.16 mg gallic acid eq/g) (*p* < 0.05). Similarly, the content of flavonoids was greater in CP (225.18 ± 0.65 mg/g) compared to PBE (143.14 ± 0.23 mg/g). The abundance of phenolic acids and flavonoids in both PBE and CP suggests they possess potential biological activities.

**Figure 1:**
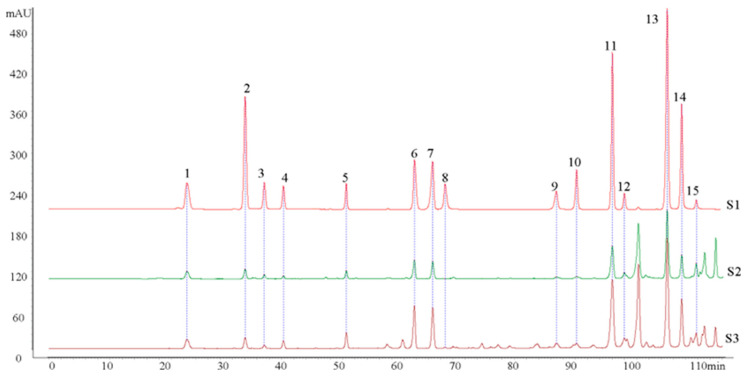
HPLC elution profiles of phytochemicals in PBE and CP. (S1: standard compounds; S2: PBE standard compounds; S3: CP standard compounds. Standard compounds: 1 Caffeic acid; 2 *p*-coumaric acid; 3 Ferulic acid; 4 Isoferulic acid; 5 3,4-dimethoxycinnamic acid; 6 Pinobanksin; 7 Naringenin; 8 Quercetin; 9 Kaempferol; 10 Apigenin; 11 Pinocembrin; 12 Benzyl caffeate; 13 Chrysin; 14 Caffeic acid phenethyl ester; 15 Galangin. PBE: poplar bud (*Populus*) extract; CP: Chinese propolis).

Fifteen major compounds, including kaempferol, apigenin, pinocembrin, benzyl caffeate, caffeic acid phenethyl ester, and galangin, were selected as standard substances for quality control purposes. An HPLC method was then employed for quality control and component analyses of PBE and CP. The results revealed a high degree of similarity in the phytochemical composition of PBE and CP (Figure 1). This observation may be attributed to the widespread cultivation of *Populus* trees in China and the preference of honeybees for *Populus* as a resin source [2]. However, the levels of specific phenolic compounds within PBE and CP differed significantly. Galangin (36.12 ± 7.12–38.72 ± 11.43 mg/g), pinocembrin (7.27 ± 2.66–13.27 ± 2.94 mg/g), and chrysin (7.92 ± 2.90–10.67 ± 2.40 mg/g) were the most abundant compounds identified in both PBE and CP. While trace amounts of isoferulic acid, pinobanksin, kaempferol, and apigenin were detected in both samples, the content of these four compounds was significantly higher in CP compared to PBE (Table 3).

### 3.2. In Vitro Antioxidant Activity

Given the reported similarity between propolis and poplar bud extract [8], CP was chosen for this comparative study. We evaluated the in vitro antioxidant activity of PBE and CP using three assays: DPPH-radical-scavenging capacity, ABTS-radical-scavenging capacity, and FRAP. The IC_50_ values for DPPH-scavenging activity were 93.04 ± 0.77 µg/mL and 93.10 ± 0.35 µg/mL for PBE and CP, respectively (Table 2). These results suggest no significant difference between PBE and CP in this assay. However, the ABTS assay revealed stronger antioxidant activity for CP (IC_50_ = 38.49 ± 0.58 µg/mL) compared to PBE (IC_50_ = 41.75 ± 1.13 µg/mL) (*p* < 0.05). Notably, both PBE and CP exhibited lower DPPH and ABTS-radical-scavenging capacities compared to VC. As expected, the in vitro antioxidant activities of both PBE and CP increased in a dose-dependent manner (Figures S1 and S2). It is well established that the FRAP assay relies on the reduction of colorless Fe^3+^-TPTZ to a blue-colored Fe^2+^-TPTZ complex by antioxidants under acidic conditions. This color change is measured at 700 nm using a spectrophotometer. This method is widely used to assess the antioxidant activity of various samples, including plants, food extracts, biological products, spices, vegetables, fruits, and essential oils [17,26]. Consistent with the other assays, the absorbance values for PBE and CP increased with increasing concentration, indicating a dose-dependent increase in reducing power (Table 2). However, both PBE and CP demonstrated a lower capacity for Fe^3+^ reduction compared to VC (Figure S3). Overall, our results demonstrate that PBE and CP possess good in vitro antioxidant activity, suggesting their potential as natural antioxidants.

### 3.3. Acute Toxicity in C. elegans

**Figure 2:**
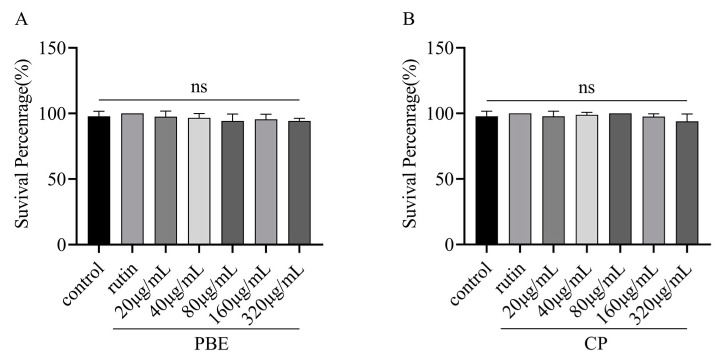
Effect of PBE (**A**) and CP (**B**) on N2 *C. elegans* viability. (After the nematodes were exposed to rutin and different concentrations of PBE and CP for 3 days, the survival rate of the nematodes was determined. The control group was 0.2% DMSO, and 36 μg/mL rutin was used as the positive control group. The concentrations of PBE and CP were 20–320 μg/mL. Data were expressed as mean ± SD of three independent experiments (*n* = 3). The “ns” means no significance. PBE: poplar bud (*Populus*) extract; CP: Chinese propolis).

Many phytochemicals are known to act as prooxidants and induce hormonal effects at specific doses. For example, *Dioscorea alata* L. tuber extracts have been shown to induce adaptive responses against helminths and extend their lifespan at low concentrations. Considering the concept of hormesis of phytochemicals, we investigated suitable concentrations of PBE and CP in this study [22]. We first determined the effect of PBE or CP on the viability of *C. elegans*. As shown in Figure 2, a range of concentrations from 20 to 320 µg/mL for 3 days did not have a significant impact on viability compared to the control group. These results suggest that PBE and CP are nontoxic and safe at these concentrations.

### 3.4. Effect of PBE and CP on Growth of C. elegans

**Figure 3:**
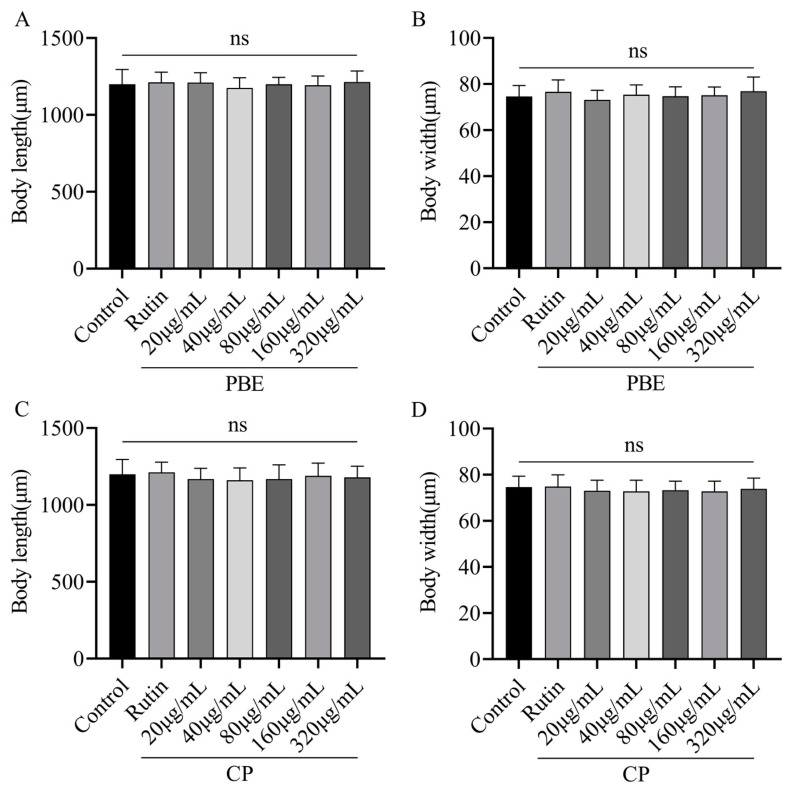
Effect of PBE or CP on the body size of *C. elegans*. ((**A**,**C**): Body length; (**B**,**D**): Body width. The control group was 0.2% DMSO, and 36 μg/mL rutin was used as the positive control group. The concentrations of PBE and CP were 20–320 μg/mL. Data were expressed as mean ± SD of three independent experiments (*n* = 20). The “ns” means no significance. PBE: poplar bud (*Populus*) extract; CP: Chinese propolis).

Nematode body length and width are established indicators of their growth and development [24]. The presence of exogenous substances within the growth environment can be reflected in the nematodes’ body size, allowing for the assessment of these substances’ influence on *C. elegans*. As Figure 3 demonstrates, none of the PBE or CP doses employed in this study (20–320 µg/mL) resulted in significant changes in body length or width. This suggests that neither PBE nor CP exhibits toxic or adverse effects on nematodes. Consequently, the maximum safe dose of 320 µg/mL was chosen for subsequent experiments.

### 3.5. Effect of PBE or CP on C. elegans Reproduction

**Figure 4:**
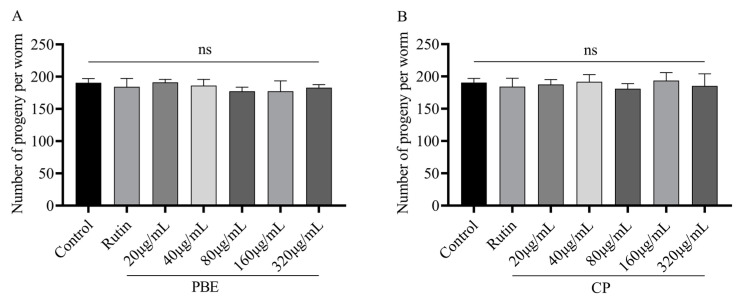
Effect of PBE (**A**) or CP (**B**) on *C. elegans* reproduction. (Total number of reproductive progenies. The control group was 0.2% DMSO, and 36 μg/mL rutin was used as the positive control group. The concentrations of PBE and CP were 20–320 μg/mL. Data were expressed as mean ± SD. The “ns” means no significance. PBE: poplar bud (*Populus*) extract; CP: Chinese propolis).

We assessed *C. elegans*’ reproductive capacity using PBE or CP to determine its impact on progeny production. The results showed that there was no significant difference in the total number of progenies between PBE, CP, and the control (Figure 4), indicating that PBE or CP had no effect on *C. elegans* progeny production.

### 3.6. PBE and CP Attenuates the Oxidative Stress Toxicity Induced by Juglone

**Figure 5:**
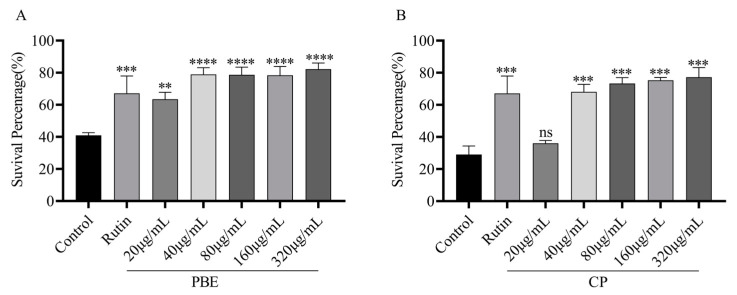
Survival rate of nematodes under oxidative stress. (The nematodes were exposed to 200 μmol/L juglone for 8 h. The control group was 0.2% DMSO, and 36 μg/mL rutin was used as the positive control group. The concentrations of PBE (**A**) and CP (**B**) were 20–320 μg/mL. The results were expressed as the mean ± SD. Compared with the control group, ns indicates no significant difference, ** *p* < 0.01, *** *p* < 0.001, and **** *p* < 0.0001. PBE: poplar bud (*Populus*) extract; CP: Chinese propolis).

Stress resistance is well established as a factor contributing to lifespan extension in *C. elegans* [27]. Juglone, a potent oxidant, elevates ROS levels in nematodes, inducing acute oxidative damage, and ultimately leading to death [28]. To determine whether PBE or CP could enhance stress tolerance, we evaluated the survival rates of nematodes under juglone induced oxidative stress. Compared to the control group, treatment with rutin and all concentrations of PBE (20–320 µg/mL) significantly improved nematode survival rates (Figure 5). Conversely, only CP concentrations of 40–320 µg/mL exerted a protective effect. Notably, both 320 µg/mL PBE and 320 µg/mL CP maintained nematode survival rates at approximately 80%. These findings suggest that PBE and CP can enhance the stress tolerance of *C. elegans*.

### 3.7. PBE and CP Reduced ROS Levels

**Figure 6:**
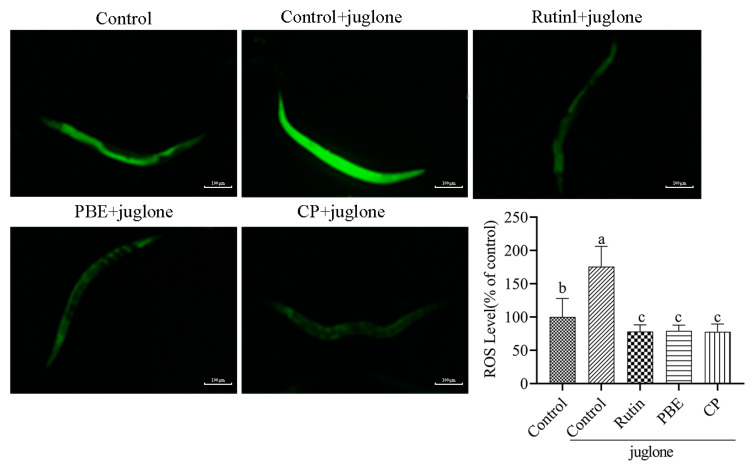
Effect of PBE and CP on intracellular ROS levels in *C. elegans*. (The nematodes were exposed to 200 μmol/L juglone for 4 h. The fluorescent probe H_2_DCFDA was used to determine the ROS content in the nematodes. The control group was 0.2% DMSO, and 36 μg/mL rutin was used as the positive control group. The concentrations of PBE and CP were 320 μg/mL. Scale bar = 100 μm. Data were expressed as mean ± SD of three independent experiments (*n* = 30). The different letters in each column indicate significant differences. PBE: poplar bud (*Populus*) extract; CP: Chinese propolis).

ROS are natural byproducts of cellular metabolism, and they possess a complex biological duality [29]. At low concentrations, ROS participate in essential physiological processes and cell signaling pathways. However, when the organism’s antioxidant defenses are overwhelmed, ROS can accumulate and inflict oxidative damage on proteins, lipids, and nucleic acids, ultimately contributing to aging and various pathologies. In this study, the H_2_DCFDA fluorescent probe was employed to quantify ROS levels in nematodes subjected to oxidative stress. As illustrated in Figure 6, compared with the control group, the ROS content of N2 nematodes exposed to juglone (control+juglone group) increased by 1.76 times, which confirmed that juglone exposure induced oxidative stress. Interestingly, compared to the control+juglone group, the ROS levels in the rutin, PBE, and CP groups were significantly reduced by 55.45%, 54.87%, and 55.64%, respectively. These findings suggest that PBE and CP, similar to other natural products [27,30], exert potent protective effects on *C. elegans* by mitigating ROS accumulation.

### 3.8. PBE and CP Enhanced Antioxidant Enzyme Activity

**Figure 7:**
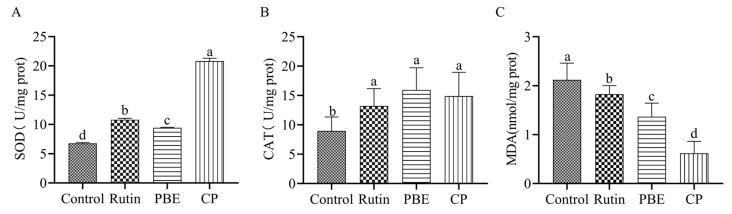
Effects of PBE and CP on SOD (**A**), CAT (**B**), and MDA (**C**) activity in *C. elegans*. (The activities of antioxidant enzymes were measured after 3 days of treatment with rutin, PBE, and CP. The control group was treated with 0.2% DMSO, and the rutin concentration was 36 μg/mL. The concentrations of PBE and CP were 320 μg/mL. Data were expressed as mean ± SD. The different letters in each column indicate significant differences. PBE: poplar bud (*Populus*) extract; CP: Chinese propolis).

Normal metabolic processes within an organism generate ROS, such as superoxide anions and hydrogen peroxide. At elevated levels, these toxic ROS can contribute to aging and organismal deterioration [31]. SOD and CAT are crucial enzymes in the antioxidant defense system. They effectively eliminate excess ROS and mitigate oxidative damage within organisms [32]. MDA, a byproduct of lipid peroxidation, acts as a potent oxidant, causing cellular damage and accelerating the aging process. Compared to the control group, rutin treatment in nematodes resulted in increased SOD and CAT activity, along with a decrease in MDA content (Figure 7). Notably, PBE treatment elevated SOD activity by 39.14%, while CP treatment displayed a more pronounced increase of 208%. Similarly, PBE and CP significantly enhanced CAT activity by 77.90% and 66.85%, respectively (Figure 7B). Furthermore, both PBE and CP mirrored the effect of rutin, significantly reducing MDA levels in nematodes (Figure 7C). These findings suggest that both PBE and CP possess antioxidant properties.

### 3.9. PBE and CP Extended the Stress Resistance Thought daf-16

**Figure 8:**
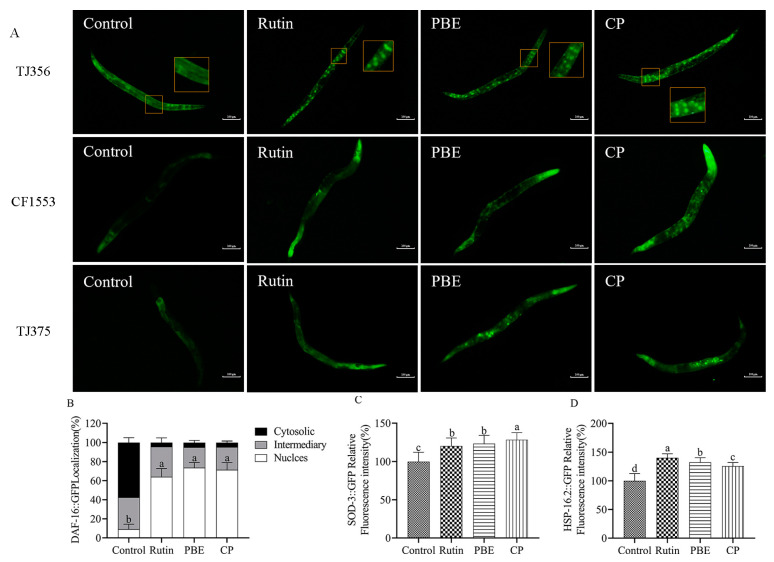
PBE and CP enhanced stress resistance through *daf-16* signaling. ((**A**): Image of the fluorescence intensity in *C. elegans* strains TJ356, CF1553, and TJ375; (**B**–**D**): Quantification of DAF-16::GFP, SOD-3::GFP, and HSP-16.2::GFP. The control group was 0.2% DMSO, and 36 μg/mL rutin was used as the positive control group. The concentrations of PBE and CP were 320 μg/mL. Scale bar = 100 μm. Results were expressed as mean ± SD. The different letters in each column indicate significant differences. PBE: poplar bud (*Populus*) extract; CP: Chinese propolis).

**Figure 9:**
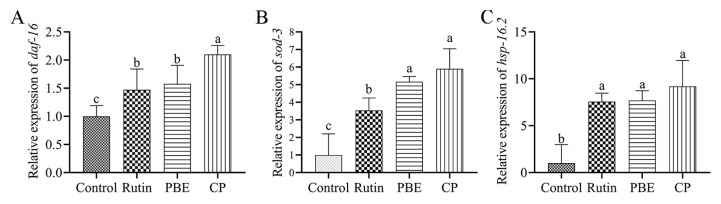
PBE and CP mediate stress resistance in a *daf-16*-dependent manner (relative mRNA levels of *daf-16* (**A**) and downstream targets (*sod-3* (**B**), *hsp-16.2* (**C**)) in *C. elegans* treated with PBE or CP. The control group was 0.2% DMSO, and 36 μg/mL rutin was used as the positive control group. The concentrations of PBE and CP were 320 μg/mL. Data were expressed as mean ± SD. The different letters in each column indicate significant differences. PBE: poplar bud (*Populus*) extract; CP: Chinese propolis).

DAF-16/FOXO, a transcription factor, plays a critical role in the insulin/IGF-1 signaling pathway (IIS pathway) of *C. elegans*, promoting longevity and stress resilience [33]. Under normal conditions, DAF-16/FOXO remains anchored in the cytoplasm through its interaction with the 14-3-3 protein [34]. However, when stimulated by certain natural products, downregulation of the DAF-2 receptor leads to DAF-16 dephosphorylation. This triggers its translocation into the nucleus, where it activates the transcription of downstream target genes like *hsp-16.2* and *sod-3*, ultimately promoting lifespan extension and stress resistance [23,35]. To investigate whether the antioxidant activities of PBE and CP might be mediated by DAF-16/FOXO translocation, the *C. elegans* strain TJ356 was treated with 320 µg/mL of each extract. Rutin, PBE, and CP treatments all resulted in a significant increase in the nuclear localization of DAF-16 transcription factors in nematodes, accompanied by a decrease in cytoplasmic localization (Figure 8A,B). PBE treatment increased the nuclear localization rate of DAF-16 from 9.28% to 73.64%. Notably, treatment with 320 µg/mL CP induced nuclear translocation in over 70% of the nematodes, with only 4.55% retaining cytoplasmic localization (Figure 8B). To further corroborate these findings, we examined the mRNA expression levels of *daf-16*. PBE treatment resulted in a 1.58-fold increase in *daf-16* expression, while CP treatment led to an even more pronounced 2.10-fold increase (Figure 9A). These results strongly suggest that PBE and CP may regulate stress resistance in *C. elegans* through modulation of DAF-16/FOXO signaling.

SOD-3, a superoxide dismutase enzyme, plays a crucial role in scavenging superoxide radicals within *C. elegans*. Under normal circumstances, *sod-3* expression is minimal; however, it is significantly upregulated in response to stress conditions, highlighting its importance in the nematode stress response [34]. Since PBE and CP treatments promoted the nuclear translocation of DAF-16 transcription factors, we investigated the expression of SOD-3, a downstream target protein of DAF-16, in *C. elegans* strain CF1553. Compared to the control group, PBE and CP treatments significantly increased the fluorescence intensity of SOD-3 in nematodes by 23.54% and 28.63%, respectively (Figure 8A,C). Furthermore, quantitative analysis revealed a 5.17-fold and 5.90-fold increase in *sod-3* expression following PBE and CP treatment, respectively (Figure 9B). These findings strongly suggest that the antioxidant activity of PBE and CP may be mediated, at least in part, by SOD-3 upregulation.

Beyond DAF-16’s role in oxidative stress response, certain heat shock proteins (HSPs) also play vital roles. For instance, *hsp-16.2*, which can be induced by oxidative stress, helps protect organisms from harsh environments [25]. Therefore, we investigated the expression of HSP-16.2 in *C. elegans* strain TJ375. Treatment with rutin, PBE, and CP all resulted in a significant increase in the fluorescence intensity of HSP-16.2::GFP, suggesting that the enhanced stress resistance observed in nematodes is associated with elevated HSP-16.2 expression (Figure 8A,D). Consistent with this observation, PBE treatment led to a 7.69-fold increase in *hsp-16.2* mRNA expression, while CP treatment caused an even greater increase of 9.19-fold (Figure 9C). In conclusion, these findings suggest that DAF-16 and its downstream target genes, including *sod-3* and *hsp-16.2*, are key factors mediating the improvement in stress resistance in *C. elegans* by PBE and CP.

### 3.10. Skn-1 Was Required for PBE and CP Mediated Oxidative Stress Resistance

**Figure 10:**
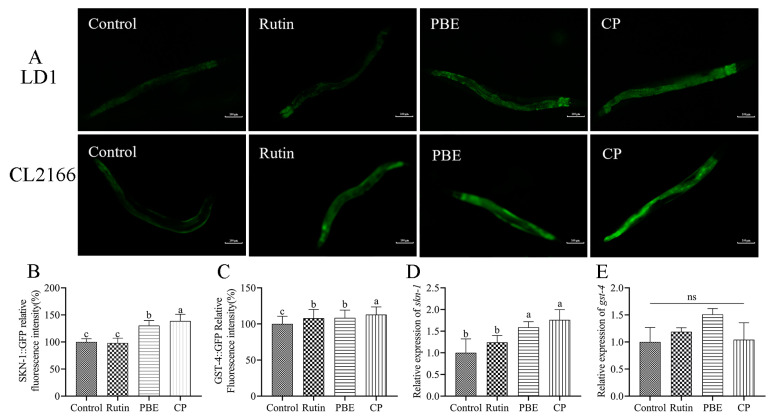
PBE and CP promote stress resistance in a *skn-1*-dependent manner. ((**A**): Image of the fluorescence intensity in *C. elegans* strains LD1 and CL2166; (**B**): Quantification of LD1::GFP; (**C**): Quantification of GST-4::GFP. (**D**,**E**): Relative mRNA levels of *skn-1* and downstream targets *gst-4* in *C. elegans* treated with PBE or CP. The control group was 0.2% DMSO, and 36 μg/mL rutin was used as the positive control group. The concentrations of PBE and CP were 320 μg/mL. Scale bar = 100 μm. Data were expressed as mean ± SD. The different letters in each column indicate significant differences. The “ns” means no significance. PBE: poplar bud (*Populus*) extract; CP: Chinese propolis).

SKN-1, like DAF-16, is a key transcription factor in the IIS pathway. It is homologous to mammalian nuclear respiration factor 2 (NRF 2) and plays a role in detoxification and the antioxidant stress response in nematodes, extending their lifespan [36]. SKN-1 is a transcription factor that acts in parallel with DAF-16 to increase the expression of phase 2 detoxification genes and trigger antioxidant responses [22]. Notably, polyphenols found in many natural plants have been shown to enhance the transcription of DAF-16 and SKN-1 within the IIS pathway, consequently influencing the expression of genes associated with the stress response [30,35]. PBE and CP increased the expression of SKN-1 in the LD1 strain, as reflected in Figure 10A,B. Meanwhile, PBE and CP treatments significantly increased *skn-1* expression, with fold-changes of 1.59 and 1.76 compared to the control group (Figure 10D), respectively. We next studied the effects of PBE and CP on GST-4 expression. While PBE and CP did upregulate *gst-4* expression, the difference was not statistically significant compared to the control group (Figure 10E). Figure 10A,C show increased GFP fluorescence intensity of GST-4 in CL2166 mutants treated with PBE or CP compared to the control group. These findings suggest that PBE and CP may promote GST-4 expression to mitigate oxidative damage. In conclusion, our data suggest that PBE and CP may exert their antioxidant effects by activating *skn-1* expression and potentially increasing the expression of its downstream target protein, GST-4.

## 4. Discussion

The botanical source of propolis significantly influences its chemical composition, leading to substantial variations between different propolis types. In this study, the characteristic components of PBE and CP, collected from the same region, were analyzed using HPLC due to ongoing debate regarding their composition. The results revealed similar components but differing content levels between CP and PBE. Galangin, pinocembrin, and chrysin emerged as the dominant chemical components in PBE, with even higher concentrations detected in CP. This difference was attributed to the diverse plant sources from which bees collect resin, as evidenced by the higher content of isoferulic acid, galangin, pinobanksin, and apigenin in CP compared to PBE. Notably, kaempferol, naringin, and quercetin were exclusively identified in PBE not detected in extracts from *Populus*. *balsamifera* buds. These findings support the notion that the dominant plant species in a given region significantly impact propolis composition. This explains the observed variations between Chinese propolis and Lithuanian propolis, as reported in previous studies [3]. Our research further confirms that CP exhibits higher levels of phenolic acids and flavonoids compared to PBE (Table 2). Additionally, Chinese propolis demonstrates a greater phenolic content and a richer profile of active ingredients when compared to Brazilian green propolis, African propolis, and Lithuanian propolis [3,37]. Plant variety, extraction techniques, and the choice of dissolving reagents are all likely contributors to these observed differences [38].

The ABTS, DPPH, and FRAP assays consistently demonstrated excellent antioxidant properties in both PBE and CP. This aligns with previous research highlighting the in vivo and in vitro antioxidant activity of various monomeric components commonly found in propolis, including rutin, quercetin, chrysin, and caffeic acid phenethyl ester [39]. These findings suggest that the total phenolic acid and flavonoid content, which is higher in CP compared to PBE, plays a key role in determining propolis’ antioxidant capacity. The lower IC_50_ values of PBE and CP compared to green propolis further underscore their superior antioxidant activity, likely due to their richer profile of phenolic acid and flavonoids [40]. However, the complexity of biological systems should be acknowledged. Natural products often contain numerous functional components that can interact synergistically within the body, making it challenging to consistently replicate in vitro results in vivo. Low doses of natural products can extend lifespans in *C. elegans* through DAF-16/FOXO3, SKN1/Nrf2 and HSF-1 signaling pathways, but high doses may be toxic and increase oxidative damage [22,25]. We used nematodes to test the toxicity of PBE and CP as stressors, finding no difference compared to the control group in terms of lethality, growth, and reproduction. The solubility of PBE or CP in NGM was influenced by the extraction methods, reagents used for extraction, and administration methods. The further investigation is needed to explore the potential toxic effects of higher doses of PBE or CP. We employed a *C. elegans* model exposed to juglone, a prooxidant known to induce oxidative stress. Juglone induces oxidative stress by promoting the generation of reactive oxygen species, damaging cell membranes and proteins, and inducing mitochondrial damage, which has harmful effects on cells and organisms [41]. As expected, juglone treatment reduced nematode survival. However, PBE and CP interventions significantly improved nematode survival rates, indicating their potent antioxidant activity in vivo. Furthermore, the effects of PBE and CP may be related to its active components such as rutin, quercetin, and phenylethyl caffeic acid [39,42]. These treatments enhanced the activity of antioxidant enzymes (SOD and CAT) while reducing MDA and ROS content within the nematodes (Figure 6 and Figure 7). These findings suggest that PBE and CP may enhance stress resistance in *C. elegans*, potentially through their own antioxidant properties or by strengthening the nematodes’ intrinsic antioxidant defense system.

Natural products can improve stress resistance in nematodes through various mechanisms. For instance, Manuka honey can improve oxidative stress indicators in AD model CL4176 *C. elegans* through HSP-16.2 and SKN-1/NRF2 pathways and delay Aβ-induced paralysis [43]. Similarly, orange extracts have been shown to activate the expression of *sek-1* and *skn-1* genes in the MAPK pathway, along with antioxidant genes like *sod-3* and *gst-4* [27]. Oleuropein enhances stress resistance and lifespan by influencing the IIS pathway and the antioxidant response factor SKN-1/NRF2 in *C. elegans* [44]. Notably, DAF-16/FOXO within the IIS pathway plays a critical role in lifespan extension and stress resistance in *C. elegans* [34]. To elucidate the specific mechanisms by which PBE and CP exert their antioxidant effects in nematodes, we investigated key factors within the IIS pathway. Following 3 days of PBE or CP treatment, we observed increased expression of *daf-16*, *sod-3*, and *hsp-16.2* genes in the nematodes (Figure 9). Furthermore, enhanced nuclear translocation of DAF-16 and the elevated expression of SOD-3::GFP and HSP-16.2::GFP fusion proteins strongly suggest that PBE and CP promote the nematodes’ endogenous antioxidant defense system through the DAF-16/FOXO pathway.

Similar to the Nrf2 transcription factor in mammals, the SKN-1 protein in *C. elegans* plays a vital role in the cellular response to stress [45]. Under stress conditions, SKN-1 accumulates in the nucleus of intestinal cells, triggering the activation of phase II detoxification genes like *gst-4*. We observed a significant increase in the expression of both *skn-1* and the SKN-1::GFP fusion protein, suggesting that PBE and CP may activate this pathway (Figure 10). Interestingly, the similar components found in PBE and CP might explain why they utilize the same signaling pathway to reduce oxidative damage in nematodes. In conclusion, our data suggest that PBE and CP exert their antioxidant effects by modulating the expression of genes (*daf-16*, *sod-3*, *hsp-16.2*, and *skn-1*) associated with the IIS pathway, alongside directly influencing antioxidant enzymes (SOD and CAT). These findings, coupled with its comparable antioxidant properties, position PBE as a promising alternative to CP. In order to confirm this result and study whether PBE and CP have anti-aging and neuroprotective effects, it is necessary to fully address the antioxidant mechanisms of PBE and CP in the presence or absence of prooxidants.

**Figure 11:**
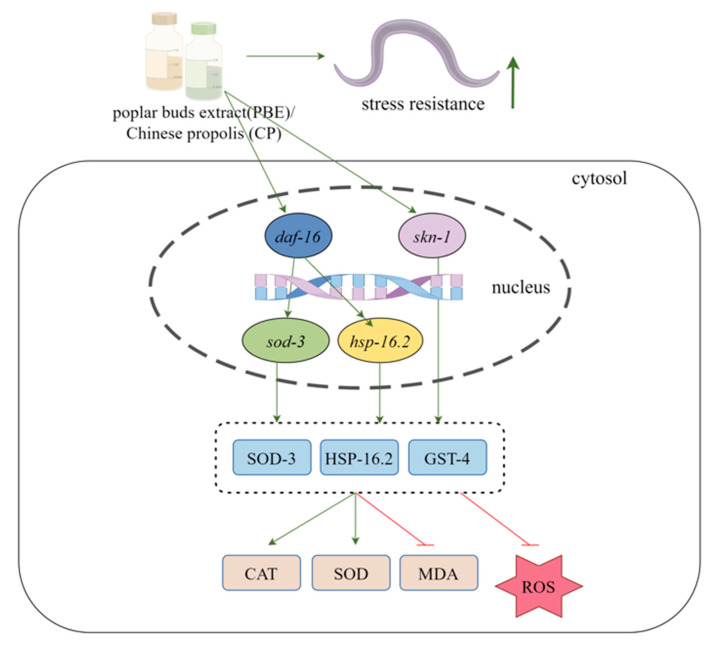
Schematic diagram showing the antioxidant mechanism of PBE and CP in vitro and in *C. elegans* (Green represents up-regulation, red represents down-regulation, arrows represent promotion, and flat heads represent inhibition; this picture was generated by Figdraw 2.0).

The findings presented in Figure 11 indicate that PBE and CP share similar pathways in increasing stress resistance within *C. elegans*.

## 5. Conclusions

This study demonstrates that PBE and CP, despite sharing similar active components, may exhibit differences in their content levels, potentially influencing their antioxidant activity (as shown in vitro assays). Furthermore, both PBE and CP were found to modulate the activity of antioxidant enzymes in nematodes. Our data suggest that PBE and CP may enhance stress resistance in *C. elegans*, possibly through the involvement of DAF-16 and SKN-1 within the IIS pathway. These findings contribute to a better understanding of the mechanisms by which poplar bud extracts and Chinese propolis exert their antioxidant effects. Further research is necessary to explore the safety and efficacy of PBE as a potential nutraceutical or pharmaceutical antioxidant.

## Figures and Tables

**Table 1:** Primer sequences.

Gene	Primer (5′-3′)
*act-1*	F: CTGTCCTCTCCCTCTACGCTTCC R: CAGTAAGATCACGTCCAGCCAAGTC
*daf-16*	F: CCACCACCATCATACCACGAGTTG R: CATTGGCTTGAAGTTAGTGCTTGGC
*sod-3*	F: ATCACTATTGCGGTTCAAGGCTCTG R: TTGCACAGGTGGCGATCTTCAAG
*skn-1*	F: GGTCTCCGTTGGCGTGATGATC R: CTGGTGGATGCTCGGTGAGTATTG
*gst-4*	F: ATGCTCGTGCTCTTGCTGAG R: GACTGACCGAATTGTTCTCCAT
*hsp-16.2*	F: AAGCGCCAAAGAAAGAAGCG R: TTCAAGTTTATTGCAGCGAACA

**Table 2:** In vitro antioxidant activity of PBE and CP.

Sample	Total Flavonoid (mg rutin/g)	Total Phenolic (mg gallic acid/g)	DPPH IC_50_ (µg/mL)	ABTS IC_50_ (µg/mL)	FRAP OD 700 nm
VC	/	/	23.29 ± 0.09 ^b^	17.61 ± 0.66 ^c^	0.38 ± 0.005
PBE	143.14 ± 0.23 ^b^	234.18 ± 0.95 ^b^	93.04 ± 0.77 ^a^	41.75 ± 1.13 ^a^	0.40 ± 0.002
CP	225.18 ± 0.65 ^a^	265.77 ± 2.16 ^a^	93.10 ± 0.35 ^a^	38.49 ± 0.58 ^b^	0.39 ± 0.003

IC_50_: concentration capable of inhibiting 50% of the free radical. The values are expressed as the mean ± SD. The different letters in each column indicate significant differences. VC was used as a control group in the in vitro antioxidant assay. The FRAP results measure the absorbance of PBE (600 µg/mL), CP (600 µg/mL), and VC (80 µg/mL) after reacting with Fe^3+^-TPTZ. Higher absorbance values correspond to greater total reducing power. VC: vitamin C; PBE: poplar bud (*Populus*) extract; CP: Chinese propolis.

**Table 3:** Major phenolic acids and flavonoids present in PBE and CP.

Compounds	Contents (mg/g of Extract)	
	PBE	CP
Caffeic acid	0.56 ± 0.20	0.62 ± 0.15
*p*-Coumaric acid	1.27 ± 0.45	1.25 ± 0.29
Ferulic acid	0.62 ± 0.22	0.49 ± 0.14
Isoferulic acid	0.51 ± 0.18 ^b^	1.26 ± 0.29 ^a^
3,4-Dimethoxycinnamate	1.34 ± 0.46	2.27 ± 0.57
Pinobanksin	3.08 ± 1.07 ^b^	5.97 ± 1.38 ^a^
Naringenin	3.79 ± 1.32	7.48 ± 1.72
Quercetin	0.10 ± 0.04	0.06 ± 0.04
Kaempferol	1.11 ± 0.44 ^b^	2.56 ± 0.57 ^a^
Apigenin	0.90 ± 0.33 ^b^	1.63 ± 0.27 ^a^
Pinocembrin	7.27 ± 2.66	13.27 ± 2.94
Benzyl caffeate	6.11 ± 2.46	7.65 ± 1.95
Chrysin	7.92 ± 2.90	10.67 ± 2.40
Caffeic acid phenethylester	2.71 ± 1.15	4.64 ± 1.01
Galangin	38.72 ± 11.43	36.12 ± 7.12

Different letters in each column indicate significant differences. The values were expressed as the mean ± SD. PBE: poplar bud (*Populus*) extract; CP: Chinese propolis.

## Data Availability

Several data, which were generated during the current study, are not publicly available, but are available from the corresponding author on reasonable request.

## Supplementary Materials

The following supporting information can be downloaded at: https://www.mdpi.com/article/10.3390/antiox13070860/s1, Figure S1: Scavenging ability of DPPH free radicals by VC, PBE, and CP. (A: VC, B: PBE, C: CP); Figure S2. Scavenging ability of ABTS free radicals by VC, PBE, and CP. (A: VC, B: PBE, C: CP); Figure S3: The reducing ability of VC, PBE, and CP. (The higher the absorbance value, the stronger the reduction ability. A: VC, B: PBE, C: CP); Figure S4: HPLC elution profiles of phytochemicals in PBE and CP. (S1: standard compounds; S2: PBE standard compounds; S3: CP standard compounds; PBE: poplar bud (Populus) extract; CP: Chinese propolis).

## Abbreviations

PBE: poplar bud (*Populus*) extract, CP: Chinese propolis, *C. elegans*: *Caenorhabditis elegans*, CAT: measuring catalase, MDA: malondialdehyde, SOD: superoxide dismutase, DPPH: 2,2-diphenyl-1-picrylhydrazy, ABTS: 2,2′-azino-bis (3-ethylbenzothiazoline-6-sulfonic acid, FFRAP: ferric-reducing antioxidant power, H_2_DCFDA: 2′,7′-dichlorofiuorescin diacetate, HPLC: high-performance liquid chromatography, ROS: reactive oxygen species, IIS pathway: insulin/IGF-1 signaling pathway
