# Supplementary material for: Poplar Bud (Populus) Extraction and Chinese Propolis Counteract Oxidative Stress in Caenorhabditis elegans via Insulin/IGF-1 Signaling Pathway
Source: Antioxidants (Basel). 2024 Jul 18;13(7):860. doi: 10.3390/antiox13070860 (PMC11274317; doi:10.3390/antiox13070860)
Supplement: Supplementary file 1 [file antioxidants-13-00860-s001.zip › antioxidants-3052954-supplementary.pdf]

Supplementary Materials

Poplar Bud (*Populus*) Extraction and Chinese Propolis Counteract Oxidative Stress in *Caenorhabditis elegans* via Insulin/IGF-1 Signaling Pathway

Shuo Wang [1,†], Chengchao Yang [2,†], Yaling Luo [1], Qingyi Chen [1], Mengyang Xu [1], Yuntao Ji [1], Xiasen Jiang [1,*] and Changqing Qu [1,*]

[1] Engineering Technology Research Center of Anti-Aging Chinese Herbal Medicine of Anhui Province, Biology and Food Engineering School, Fuyang Normal University, Fuyang 236000, China; wangswage@163.com (S.W.); lyl1358211941@163.com (Y.L.); chenqingyi971030@163.com (Q.C.); m19577308643@163.com (M.X.); jiyuntao@163.com (Y.J.)

[2] Liaoning Provincial Institute of Poplar, Gaizhou 115200, China; woodbreeding@126.com

* Correspondence: jxs@fynu.edu.cn (X.J.); qucq518@163.com (C.Q.)

† These authors contributed equally to this work.

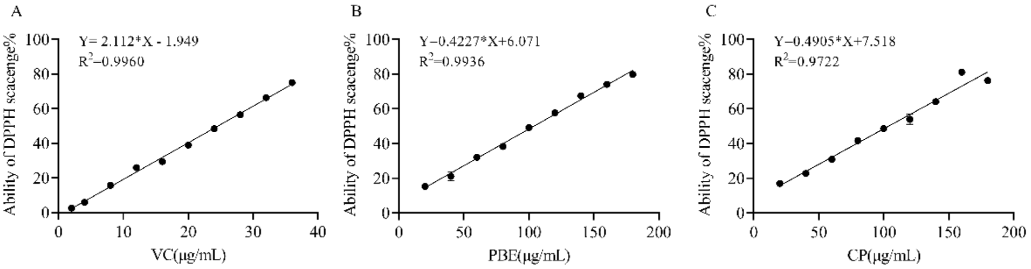

Figure S1. Scavenging ability of DPPH free radicals by VC, PBE and CP. ((**A**): VC: vitamin C, (**B**): PBE-poplar bud (*Populus*) extract; (**C**): CP: Chinese propolis).

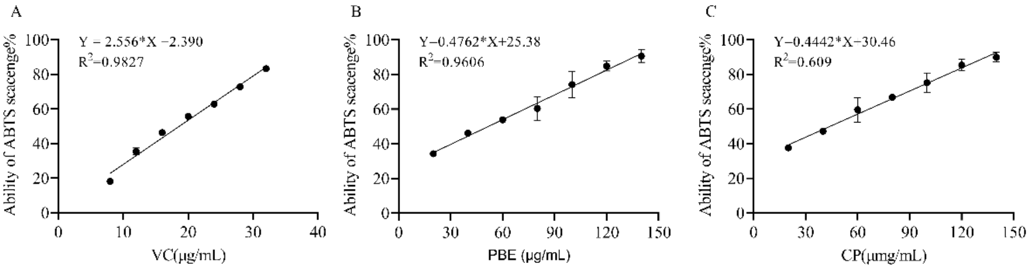

Figure S2. Scavenging ability of ABTS free radicals by VC, PBE and CP. ((**A**): VC: vitamin C, (**B**): PBE-poplar bud (*Populus*) extract; (**C**): CP: Chinese propolis).

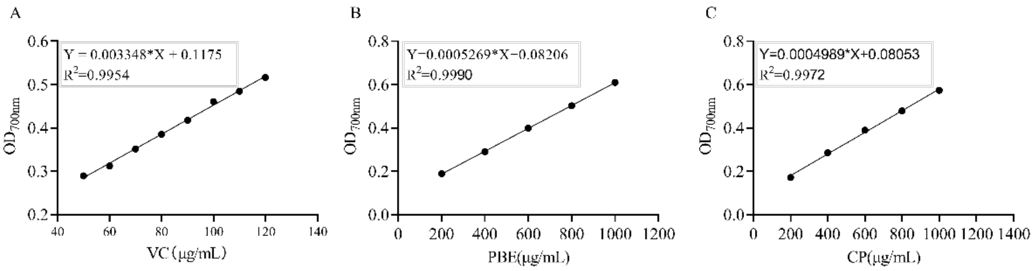

Figure S3. The reducing ability of VC, PBE and CP. (The higher the absorbance value, the stronger the reduction ability. ((**A**): VC: vitamin C, (**B**): PBE-poplar bud (*Populus*) extract; (**C**): CP: Chinese propolis).

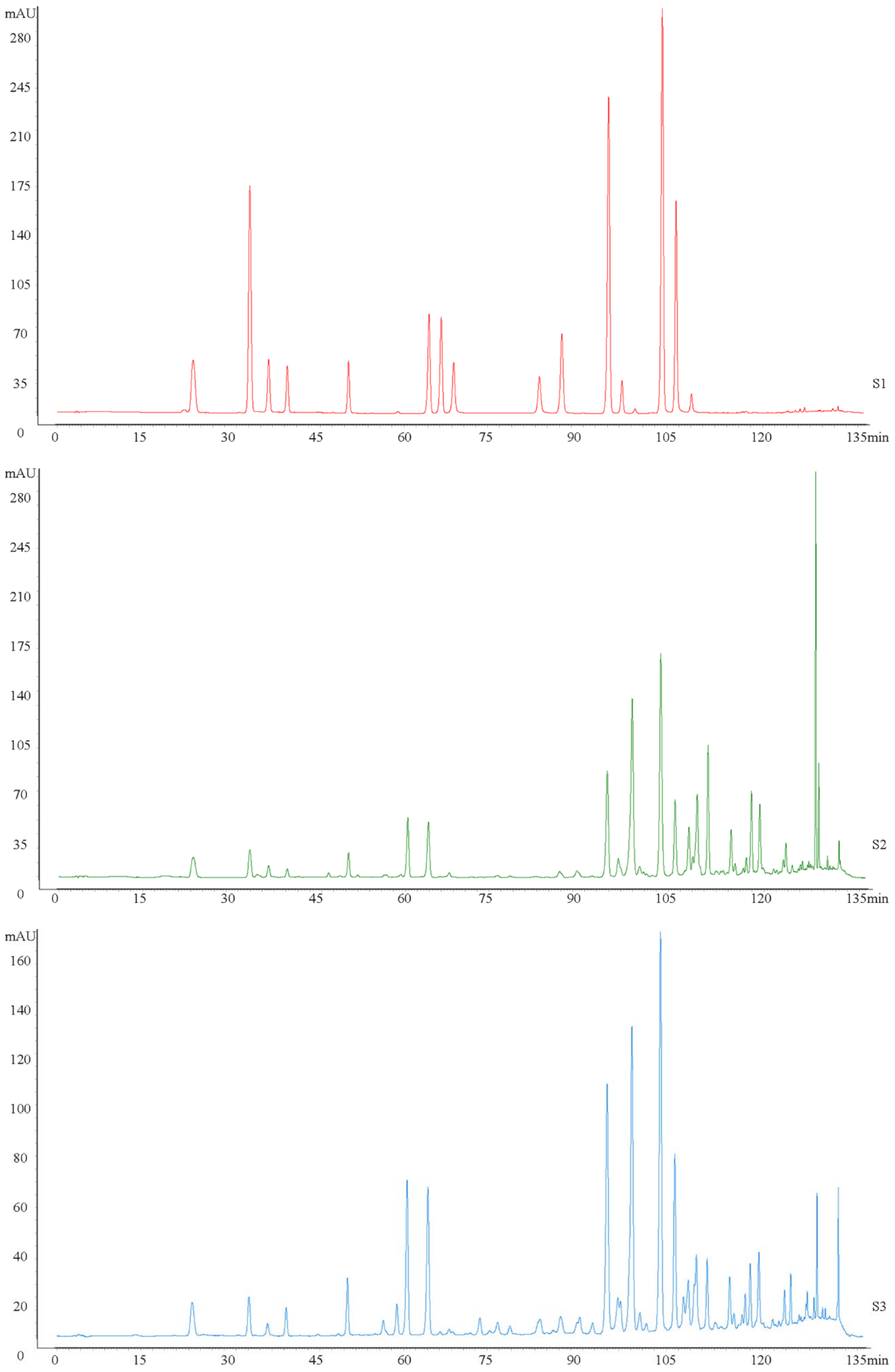

Figure S4. HPLC elution profiles of phytochemicals in PBE and CP. ((**S1**): standard compounds; (**S2**): PBE standard compounds; (**S3**): CP standard compounds. PBE: poplar bud (*Populus*) extract; CP: Chinese propolis).
